# Impact of obstructive sleep apnea on inpatient outcomes of COVID-19: a propensity-score matching analysis of the US Nationwide Inpatient Sample 2020

**DOI:** 10.3389/fmed.2025.1472176

**Published:** 2025-03-20

**Authors:** Wei Du, Hong Xu, Yunqi Chang, Biying Feng, Qiong Wang, Weifeng Li

**Affiliations:** ^1^Department of Respiratory Medicine, General Hospital of Southern Theater Command, Guangzhou, China; ^2^Department of Disease Control and Prevention, General Hospital of Southern Theater Command, Guangzhou, China

**Keywords:** obstructive sleep apnea (OSA), COVID-19, Nationwide Inpatient Sample (NIS), in-patient outcome, mortality, respiratory failure

## Abstract

**Background:**

Obstructive sleep apnea (OSA) is associated with health complications, but its impact on COVID-19 outcomes is not known. This study investigated the association between OSA and outcomes of hospitalized COVID-19 patients.

**Methods:**

The Nationwide Inpatient Sample 2020 was searched for adults hospitalized for COVID-19. The outcomes of interest were in-hospital mortality, non-routine discharge, prolonged length of stay (LOS), and complications. Patients with OSA were matched to those without OSA in a 1:4 ratio using propensity score matching (PSM) according to age, sex, and major comorbidities.

**Results:**

After PSM, there were 54,900 adult COVID-19 patients consisting of 10,980 with OSA and 43,920 without OSA. The mean age was 63.2 years and 62.8% were male. Patients with OSA had higher odds of respiratory failure (adjusted OR [aOR] = 1.20, 95% confidence interval [CI]: 1.14–1.25), heart failure (aOR = 1.71, 95% CI: 1.60–1.82), and arrhythmias (aOR = 1.18, 95% CI: 1.08–1.30). Conversely, OSA was associated with lower odds of cerebrovascular accidents (CVAs) (aOR = 0.71, 95% CI: 0.62–0.81, *p* < 0.001), and a reduced likelihood of in-hospital mortality among patients ≥70 years old (aOR = 0.82, 95% CI: 0.75–0.89, *p* < 0.001) and males (aOR = 0.79, 95% CI: 0.72–0.88, *p* < 0.001), but not females.

**Conclusion:**

OSA is associated with higher risks of respiratory failure, heart failure, and arrhythmias in patients hospitalized for COVID-19. However, patients with OSA who are ≥70 years old and those who are male are less likely to have CVAs and in-hospital mortality. These findings underscore the complex relationship between OSA and COVID-19. As the study focused on hospitalized patients, the findings may not apply to mild or asymptomatic COVID-19 cases. Future research should include community-based cohorts and prospective studies to better understand this association.

## 1 Introduction

Obstructive sleep apnea (OSA) affects a wide range of people with varying severity, and the prevalence in adults ranges from 9 to 38%, with higher rates in older individuals and men (1). OSA is linked to hypertension, cardiovascular disease (CVD), stroke, and various other health complications, resulting in a significant economic burden (2, 3). Key risk factors include obesity, aging, male sex, and some lifestyle factors such as alcohol and smoking (4). Treatment options for OSA prominently include continuous positive airway pressure (CPAP) therapy. Despite its effectiveness, achieving consistent treatment compliance presents a significant challenge in OSA management (5).

The global COVID-19 pandemic, caused by the SARS-CoV-2 virus, has resulted in severe health complications such as pneumonia and acute respiratory distress syndrome (ARDS) for many affected individuals (6, 7). Factors that are associated with worse outcomes for patients infected with COVID-19 include being male, age over 65 years, smoking, hypertension, diabetes, CVD, and respiratory diseases (8).

Studies of the impact of OSA on COVID-19 outcomes, especially regarding mortality, have shown mixed results (9). While some studies suggest higher rates of adverse outcomes such as increased readmissions, the need for mechanical ventilation, and mortality in OSA patients, others have not found a significant impact of OSA on mortality rates among COVID-19 patients (10, 11). This inconsistency highlights the need for more research to fully understand the role of OSA in COVID-19 outcomes, particularly from a population-wide perspective.

Therefore, this study aimed to investigate the potential impact of OSA in patients hospitalized for a COVID-19 infection, and thus help to clarify inconsistencies in the current literature. This could provide valuable insights into the management and treatment strategies of patients affected by both conditions.

## 2 Methods

### 2.1 Data source

Data were obtained from the 2020 Nationwide Inpatient Sample (NIS), a database created by the Healthcare Cost and Utilization Project (HCUP) and maintained by the Agency for Healthcare Research and Quality (AHRQ). The NIS database includes a 20% stratified sample of inpatient admissions from US hospitals, encompassing various patient demographics, diagnoses, procedures, and outcomes.

### 2.2 Study design and ethical considerations

This retrospective study utilized de-identified data from the NIS in accordance with HCUP’s data-use agreement. The Institutional Review Board of the General Hospital of Southern Theater Command granted an exemption from further approval and informed consent requirements, as only de-identified data were used and did not directly involve patients.

Patients older than 18 years hospitalized for a COVID-19 infection were eligible for inclusion. Exclusion criteria were: (1) missing information on sex or main study outcomes; (2) chronic obstructive pulmonary disease (COPD); and (3) missing dataset weight values. The International Classification of Diseases, Ninth Revision, and Tenth Revision, Clinical Modification (ICD-9-CM and ICD-10-CM) codes identified all diagnoses and procedures, detailed in Supplementary Table S1.

### 2.3 Outcomes of interest and study variables

The primary outcomes analyzed were in-hospital mortality, non-routine discharge (i.e., discharged to long-term care facilities), prolonged LOS (defined as ≥75th percentile LOS in the study population), and complications including arrhythmias, heart failure, venous thromboembolism (VTE), mechanical ventilation, respiratory failure, disseminated intravascular coagulation, encephalitis, gastrointestinal hemorrhage, acute pancreatitis, acute cholecystitis, and hemophagocytic lymph histiocytosis (HLH). Demographic variables included age, sex, ethnicity, smoking status, obesity, household income, insurance status, and whether the admission occurred on a weekend. The Charlson Comorbidity Index (CCI) was used to quantify the overall severity of comorbid conditions. Hospital-related characteristics (bed size, location/teaching status, and region) were also included.

### 2.4 Statistical analysis

The NIS database includes a 20% sample of annual inpatient admissions in the United States. Analyses used weighted samples (DISCWT 2020), with stratum (NIS_STRATUM) and cluster (HOSPID) to generate national estimates. Statistical analyses were conducted using SAS version 9.4, employing the SURVEY procedure. Descriptive statistics were presented as numbers (n) and weighted percentages (%), or means and standard errors (SE). Categorical data were analyzed with PROC SURVEYFREQ using the Rao-Scott chi-square test, while continuous data were assessed with PROC SURVEYREG, fitting linear models for survey data.

Patients with OSA were matched to those without OSA using propensity score matching (PSM) in a 1:4 ratio, adjusted for age, sex, and major comorbidities. Univariate and multivariable logistic regression analyses were performed with PROC SURVEYLOGISTIC to determine associations between study variables and outcomes, including in-hospital mortality, prolonged LOS, and complications. Results were reported as odds ratios (OR) with 95% confidence intervals (CI). Multivariable analyses adjusted for variables significant in the univariate analysis. All *p*-values were two-sided, with <0.05 considered statistically significant.

## 3 Results

### 3.1 Patient selection

**Figure 1:**
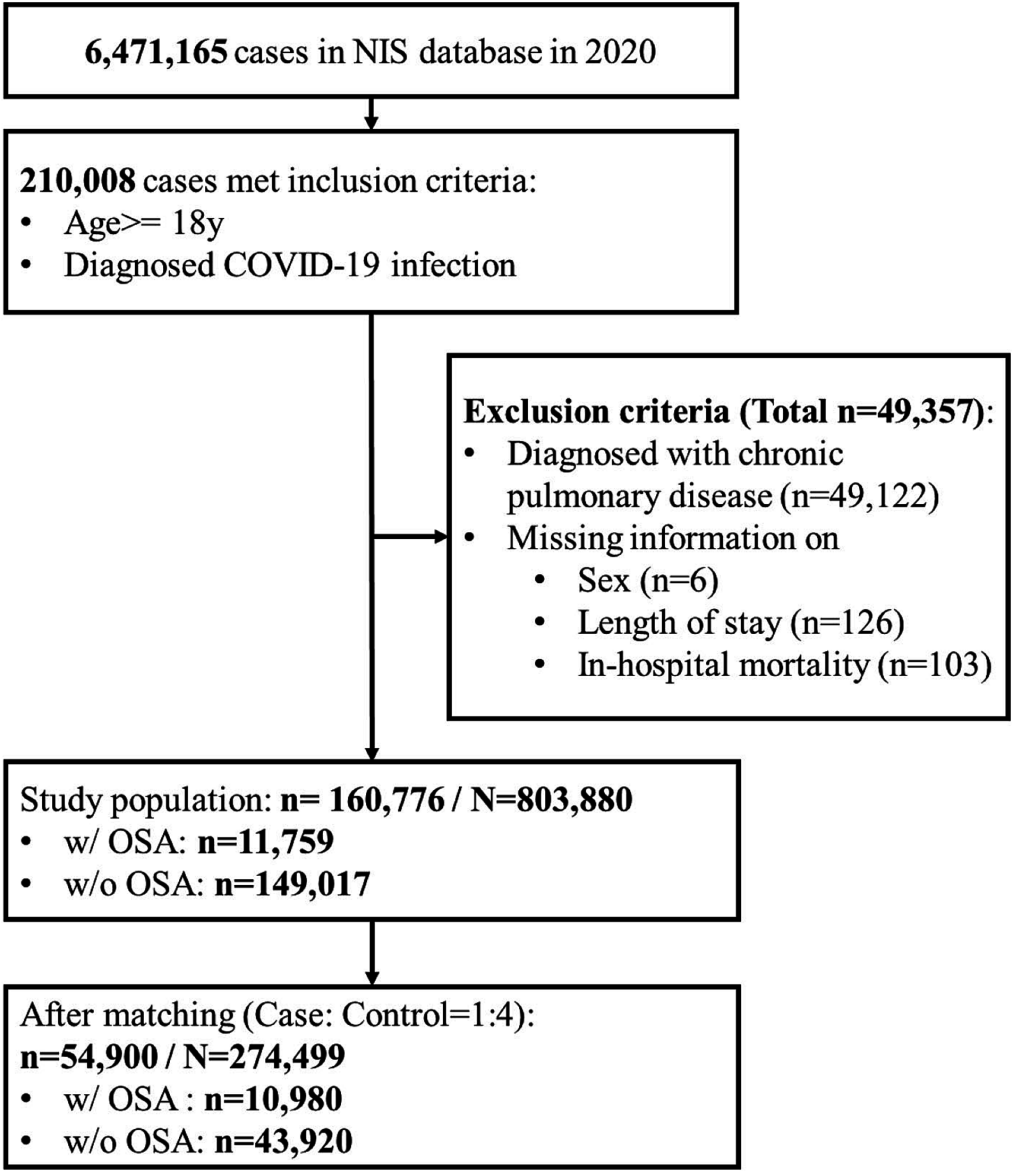
Flow diagram of patient selection.

The selection process for the study population is illustrated in Figure 1. The study identified a total of 210,008 adults ≥18 years old hospitalized for COVID-19 infection in the NIS database in 2020. Patients who were diagnosed with COPD (*n* = 49,122), or with missing information on sex (*n* = 6), LOS (*n* = 126), and in-hospital mortality status (*n* = 103) were excluded and data from 160,776 patients remained. After matching, 10,980 patients with OSA and 43,920 without OSA were included in the analysis. This sample represents a total of 27,4,499 hospitalizations in the whole US after applying the weighting method provided by the NIS dataset.

### 3.2 Characteristics of the study population

The characteristics of patients before and after PSM are shown in Table 1. In the entire study population, those without OSA were significantly older (64.1 ± 0.1 vs. 62.6 ± 0.1 years, *p* < 0.001). Patients with OSA exhibited higher proportions of ischemic heart disease (26.9% vs. 19.1%, *p* < 0.001), atrial fibrillation (10.2% vs. 7.9%, *p* < 0.001), hypertension (69.5% vs.59.3%, *p* < 0.001), diabetes (51.9% vs. 39.8%, *p* < 0.001), obesity (57.4% vs. 22.9%, *p* < 0.001), and chronic kidney disease (25.3% vs.18.8%, *p* < 0.001). After matching, there still existed differences between the 2 study groups in the distribution of sex, household income, insurance status, diabetes, positive airway pressure (PAP) use, hospital bed size, hospital location/teaching status, and hospital region (Table 1).

**Table 1:** Characteristics and in-hospital outcomes of patients hospitalized for COVID-19 with or without OSA, before and after propensity score matching.

Characteristics	Before matching				After matching			
	Total	OSA	No OSA	*p*-value	Total	OSA	No OSA	*p*-value
	(*n* = 160,776)	(*n* = 11,759)	(*n* = 149,017)		(*n* = 54,900)	(*n* = 10,980)	(*n* = 43,920)	
Age, years	64.0 ± 0.1	62.6 ± 0.1	64.1 ± 0.1	**<0.001**	63.2 ± 0.1	62.7 ± 0.2	63.3 ± 0.1	**<0.001**
18–29	4,003 (2.5)	150 (1.3)	3,853 (2.6)	**<0.001**	739 (1.3)	150 (1.4)	589 (1.3)	0.635
30–49	9,241 (5.7)	567 (4.8)	8,674 (5.8)		2,851 (5.2)	561 (5.1)	2,290 (5.2)	
40–59	18,243 (11.3)	1,395 (11.9)	16,848 (11.3)		6,430 (11.7)	1,339 (12.2)	5,091 (11.6)	
50–69	29,314 (18.2)	2,583 (22.0)	26,731 (17.9)		11,689 (21.3)	2,340 (21.3)	9,349 (21.3)	
60–69	35,434 (22.0)	3,094 (26.3)	32,340 (21.7)		13,941 (25.4)	2,779 (25.3)	11,162 (25.4)	
70+	64,541 (40.1)	3,970 (33.8)	60,571 (40.6)		19,250 (35.1)	3,811 (34.7)	15,439 (35.2)	
Sex				**<0.001**				**0.036**
Male	88,338 (54.9)	7,751 (65.9)	80,587 (54.1)		34,478 (62.8)	6,993 (63.7)	27,485 (62.6)	
Female	72,438 (45.1)	4,008 (34.1)	68,430 (45.9)		20,422 (37.2)	3,987 (36.3)	16,435 (37.4)	
Race				**<0.001**				0.982
White	77,259 (48.1)	7,192 (61.2)	70,067 (47.0)		32,460 (59.1)	6,487 (59.1)	25,973 (59.1)	
Black	28,656 (17.8)	2,189 (18.6)	26,467 (17.8)		10,560 (19.2)	2,124 (19.3)	8,436 (19.2)	
Hispanic	35,765 (22.2)	1,439 (12.2)	34,326 (23.0)		7,256 (13.2)	1,439 (13.1)	5,817 (13.2)	
Other/unknown	19,096 (11.9)	939 (8.0)	18,157 (12.2)		4,624 (8.4)	930 (8.5)	3,694 (8.4)	
Household income				**<0.001**				**<0.001**
Quartile1	53,729 (33.9)	3,427 (29.5)	50,302 (34.3)		17,955 (33.2)	3,217 (29.7)	14,738 (34.1)	
Quartile2	43,454 (27.5)	3,459 (29.8)	39,995 (27.3)		15,385 (28.5)	3,236 (29.9)	12,149 (28.1)	
Quartile3	34,917 (22.1)	2,795 (24.1)	32,122 (21.9)		12,120 (22.4)	2,576 (23.8)	9,544 (22.1)	
Quartile4	26,172 (16.5)	1,931 (16.6)	24,241 (16.5)		8,613 (15.9)	1,811 (16.7)	6,802 (15.7)	
Missing	2,504	147	2,357		817	136	681	
Insurance status				**<0.001**				**<0.001**
Medicare/Medicaid	98,495 (61.4)	6,777 (57.7)	91,718 (61.7)		32,187 (58.7)	6,373 (58.1)	25,814 (58.9)	
Private including HMO	47,328 (29.5)	4,225 (36.0)	43,103 (29.0)		18,172 (33.2)	3,903 (35.6)	14,269 (32.5)	
Self-pay/no-charge/other	14,629 (9.1)	741 (6.3)	13,888 (9.3)		4,448 (8.1)	689 (6.3)	3,759 (8.6)	
Missing	324	16	308		92	14	78	
Smoking				**<0.001**				0.296
No	125,000 (77.7)	8,419 (71.6)	116,581 (78.2)		39,690 (72.3)	7,985 (72.7)	31,705 (72.2)	
Yes	35,776 (22.3)	3,340 (28.4)	32,436 (21.8)		15,210 (27.7)	2,995 (27.3)	12,215 (27.8)	
Comorbidity								
Influenza A & B	483 (0.3)	20 (0.2)	463 (0.3)	**0.007**	85 (0.2)	20 (0.2)	65 (0.1)	0.418
Ischemic Heart Disease	31,687 (19.7)	3,164 (26.9)	28,523 (19.1)	**<0.001**	13,931 (25.4)	2,714 (24.7)	11,217 (25.5)	0.079
Atrial fibrillation	13,030 (8.1)	1,203 (10.2)	11,827 (7.9)	**<0.001**	5,237 (9.5)	1,074 (9.8)	4,163 (9.5)	0.333
Hypertension	96,511 (60.0)	8,176 (69.5)	88,335 (59.3)	**<0.001**	37,622 (68.5)	7,483 (68.2)	30,139 (68.6)	0.34
Diabetes	65,411 (40.7)	6,103 (51.9)	59,308 (39.8)	**<0.001**	27,796 (50.6)	5,448 (49.6)	22,348 (50.9)	**0.019**
Obesity	40,929 (25.5)	6,745 (57.4)	34,184 (22.9)	**<0.001**	29,722 (54.1)	5,966 (54.3)	23,756 (54.1)	0.674
Chronic kidney disease	30,978 (19.3)	2,970 (25.3)	28,008 (18.8)	**<0.001**	13,276 (24.2)	2,605 (23.7)	10,671 (24.3)	0.818
Severe Liver disease	844 (0.5)	55 (0.5)	789 (0.5)	0.405	222 (0.4)	53 (0.5)	169 (0.4)	0.6
Rheumatic disease	4,017 (2.5)	375 (3.2)	3,642 (2.4)	**<0.001**	1,677 (3.1)	339 (3.1)	1,338 (3.0)	0.418
Any malignancy	5,235 (3.3)	359 (3.1)	4,876 (3.3)	0.205	1,677 (3.1)	344 (3.1)	1,333 (3.0)	0.079
CCI				**<0.001**				**0.005**
0	58,341 (36.3)	3,201 (27.2)	55,140 (37.0)		15,931 (29.0)	3,164 (28.8)	12,767 (29.1)	
1	44,331 (27.6)	3,190 (27.1)	41,141 (27.6)		15,492 (28.2)	2,983 (27.2)	12,509 (28.5)	
2	20,188 (12.6)	1,663 (14.1)	18,525 (12.4)		7,368 (13.4)	1,564 (14.2)	5,804 (13.2)	
3+	37,916 (23.6)	3,705 (31.5)	34,211 (23.0)		16,109 (29.3)	3,269 (29.8)	12,840 (29.2)	
PAP use				**<0.001**				**<0.001**
No	156,153 (97.1)	10,696 (91.0)	145,457 (97.6)		52,605 (95.8)	10,014 (91.2)	42,591 (97.0)	
Yes	4,623 (2.9)	1,063 (9.0)	3,560 (2.4)		2,295 (4.2)	966 (8.8)	1,329 (3.0)	
Hospital bed size				**<0.001**				**<0.001**
Small	41,343 (25.7)	2,827 (24.0)	38,516 (25.8)		13,917 (25.3)	2,652 (24.2)	11,265 (25.6)	
Medium	46,510 (28.9)	3,204 (27.2)	43,306 (29.1)		15,509 (28.2)	2,981 (27.1)	12,528 (28.5)	
Large	72,923 (45.4)	5,728 (48.7)	67,195 (45.1)		25,474 (46.4)	5,347 (48.7)	20,127 (45.8)	
Hospital location/ teaching status				**0.025**				**0.003**
Rural	18,328 (11.4)	1,372 (11.7)	16,956 (11.4)		6,655 (12.1)	1,284 (11.7)	5,371 (12.2)	
Urban nonteaching	30,982 (19.3)	2,105 (17.9)	28,877 (19.4)		10,504 (19.1)	1,955 (17.8)	8,549 (19.5)	
Urban teaching	111,466 (69.3)	8,282 (70.4)	103,184 (69.2)		37,741 (68.7)	7,741 (70.5)	30,000 (68.3)	
Hospital region				**<0.001**				**<0.001**
Northeast	28,356 (17.6)	1,588 (13.5)	26,768 (18.0)		8,677 (15.8)	1,525 (13.9)	7,152 (16.3)	
South	35,276 (21.9)	3,929 (33.4)	31,347 (21.0)		14,310 (26.1)	3,642 (33.2)	10,668 (24.3)	
Midwest	68,485 (42.6)	4,396 (37.4)	64,089 (43.0)		23,479 (42.8)	4,093 (37.3)	19,386 (44.1)	
West	28,659 (17.8)	1,846 (15.7)	26,813 (18.0)		8,434 (15.4)	1,720 (15.7)	6,714 (15.3)	

OSA, obstructive sleep apnea; PAP, positive airway pressure; HMO, Health Maintenance Organization; CCI, Charlson Comorbidity Index.

Continuous variables are presented as mean ± SE; categorical variables are presented as unweighted counts (weighted percentage).

*p-values < 0.05 are shown in bold.*

### 3.3 Outcomes

The in-hospital outcomes between patients with and without OSA are summarized in Table 2. After matching, patients with OSA exhibited significantly higher percentages of complications including arrhythmias (5.8% vs. 4.7%), heart failure (20.8% vs. 15.1%), and respiratory failure (64.7% vs. 59.6%), compared to those without OSA. In-hospital mortality and prolonged LOS were not different between patients with and without OSA (Table 2).

**Table 2:** In-hospital outcomes of patients hospitalized for COVID-19 with and without OSA, before and after propensity score matching.

Characteristics	Before matching				After matching			
	Total	OSA	No OSA	*p*-value	Total	OSA	No OSA	*p*-value
	(*n* = 160,776)	(*n* = 11,759)	(*n* = 1,49,017)		(*n* = 54,900)	(*n* = 10,980)	(*n* = 43,920)	
In-hospital mortality	17,189 (10.7)	1,175 (10.0)	16,014 (10.7)	**0.016**	5,905 (10.8)	1,094 (10.0)	4,811 (11.0)	**0.004**
Prolonged LOS, days^a,b^	27,608 (19.2)	2,274 (21.5)	25,334 (19.0)	**<0.001**	10,044 (20.5)	2,102 (21.3)	7,942 (20.3)	**0.042**
Complications, any	110,263 (68.6)	9,191 (78.2)	101,072 (67.8)	**<0.001**	40,286 (73.4)	8,564 (78.0)	31,722 (72.2)	**<0.001**
Arrhythmia	7,125 (4.4)	685 (5.8)	6,440 (4.3)	**<0.001**	2,709 (4.9)	634 (5.8)	2,075 (4.7)	**<0.001**
Heart failure	20,514 (12.8)	2,498 (21.2)	18,016 (12.1)	**<0.001**	8,908 (16.2)	2,279 (20.8)	6,629 (15.1)	**<0.001**
CVA	5,198 (3.2)	276 (2.3)	4,922 (3.3)	**<0.001**	1,764 (3.2)	262 (2.4)	1,502 (3.4)	**<0.001**
VTE	7,502 (4.7)	602 (5.1)	6,900 (4.6)	**0.019**	2,711 (4.9)	571 (5.2)	2,140 (4.9)	0.171
Mechanical ventilation	14,787 (9.2)	1,290 (11.0)	13,497 (9.1)	**<0.001**	5,793 (10.6)	1,196 (10.9)	4,597 (10.5)	0.225
Respiratory failure	91,356 (56.8)	7,614 (64.8)	83,742 (56.2)	**<0.001**	33,285 (60.6)	7,103 (64.7)	26,182 (59.6)	**<0.001**
Disseminated intravascular coagulation	392 (0.2)	29 (0.2)	363 (0.2)	0.948	126 (0.2)	26 (0.2)	100 (0.2)	0.857
Encephalitis/meningitis	133 (0.1)	7 (0.1)	126 (0.1)	0.369	37 (0.1)	7 (0.1)	30 (0.1)	0.871
Gastrointestinal hemorrhage	1,540 (1.0)	112 (1.0)	1,428 (1.0)	0.951	543 (1.0)	108 (1.0)	435 (1.0)	0.949
Acute pancreatitis	572 (0.4)	33 (0.3)	539 (0.4)	0.144	167 (0.3)	32 (0.3)	135 (0.3)	0.778
Acute cholecystitis	126 (0.1)	8 (0.1)	118 (0.1)	0.677	38 (0.1)	8 (0.1)	30 (0.1)	0.869
HLH	107 (0.1)	10 (0.1)	97 (0.1)	0.448	41 (0.1)	10 (0.1)	31 (0.1)	0.537

OSA, obstructive sleep apnea; LOS, length of hospital stay; AMI, acute myocardial infarction; CVA, cerebral vascular accident; VTE, venous thromboembolism; HLH, hemophagocytic lymph histiocytosis; PAP, positive airway pressure.

Continuous variables are presented as mean ± SE; categorical variables are presented as unweighted counts (weighted percentage).

^aExcluding patients who died in the hospital.^

^bLOS > 9 days.^

*p-values < 0.05 are shown in bold.*

### 3.4 Associations between OSA and outcomes

The associations between OSA and outcomes are shown in Table 3. Patients with OSA had higher odds of respiratory failure (adjusted OR [aOR] = 1.20, 95% confidence interval [CI]: 1.14–1.25), heart failure (aOR = 1.71, 95% CI: 1.60–1.82), and arrhythmias (aOR = 1.18, 95% CI: 1.08–1.30). Conversely, OSA was associated with lower odds of cerebrovascular accidents (CVAs) (aOR = 0.71, 95% CI: 0.62–0.81), and a reduced likelihood of in-hospital mortality (aOR = 0.83, 95% CI: 0.77–0.90) (Table 3).

**Table 3:** Associations between OSA and outcomes.

Variables	OSA vs. no OSA			
	Univariate		Multivariable	
	OR (95% CI)	*p*-value	aOR (95% CI)	*p*-value
In-hospital mortality	0.90 (0.84, 0.97)	**0.004**	0.83 (0.77, 0.90)	**<0.001**
Prolonged LOS, days^a,b,d,e^	1.06 (1.00, 1.12)	**0.042**	0.98 (0.92, 1.04)	0.476
Complications, any^c^	1.36 (1.29, 1.44)	**<0.001**	1.30 (1.23, 1.37)	**<0.001**
Arrhythmia	1.24 (1.13, 1.35)	**<0.001**	1.18 (1.08, 1.30)	**<0.001**
Heart failure	1.47 (1.40, 1.56)	**<0.001**	1.71 (1.60, 1.82)	**<0.001**
CVA	0.69 (0.61, 0.79)	**<0.001**	0.71 (0.62, 0.81)	**<0.001**
VTE	1.07 (0.97, 1.18)	0.172	0.99 (0.90, 1.10)	0.918
Mechanical ventilation	1.05 (0.97, 1.12)	0.225	0.96 (0.89, 1.04)	0.307
Respiratory failure	1.24 (1.18, 1.30)	**<0.001**	1.20 (1.14, 1.25)	**<0.001**
Disseminated intravascular coagulation	1.04 (0.68, 1.60)	0.855	0.97 (0.63, 1.51)	0.893
Encephalitis/meningitis	0.93 (0.40, 2.16)	0.872	0.91 (0.41, 2.01)	0.818
Gastrointestinal hemorrhage	0.99 (0.80, 1.23)	0.949	0.98 (0.79, 1.21)	0.838
Acute pancreatitis	0.95 (0.65, 1.37)	0.778	0.98 (0.67, 1.44)	0.934
Acute cholecystitis	1.07 (0.50, 2.30)	0.869	0.99 (0.44, 2.21)	0.977
HLH	1.29 (0.57, 2.91)	0.537	1.02 (0.46, 2.27)	0.959

OSA, obstructive sleep apnea; LOS, length of hospital stay; AMI, acute myocardial infarction; CVA, cerebral vascular accident; VTE, venous thromboembolism; HLH, hemophagocytic lymph histiocytosis; PAP, positive airway pressure; OR, odds ratio; aOR, adjusted odds ratio; CI, confidence interval.

Multivariable regression was adjusted for variables that were significant in univariate regression model. *p*-values < 0.05 are shown in bold.

^aAdjusted for age group, sex, PAP use, race, household income, insurance status, smoking, ischemic heart disease, atrial fibrillation, hypertension, diabetes, obesity, chronic kidney disease, severe liver disease, rheumatic disease, any malignancy, hospital bed size, hospital location/teaching status and hospital region.^

^bAdjusted for age group, sex, PAP use, race, household income, insurance status, smoking, ischemic heart disease, atrial fibrillation, hypertension, diabetes, obesity, chronic kidney disease, severe liver disease, hospital bed size, hospital location/teaching status and hospital region.^

^cAdjusted for age group, sex, PAP use, race, insurance status, smoking, ischemic heart disease, atrial fibrillation, hypertension, diabetes, obesity, chronic kidney disease, severe liver disease, hospital bed size, hospital location/teaching status and hospital region.^

^dExcluding patients who died in the hospital.^

^eLOS > 9 days.^

### 3.5 Stratified analysis

Stratified analyses were carried out to assess whether age, sex, and obesity moderated the associations between OSA and COVID-19 outcomes (Tables 4, 5). OSA was significantly associated with a lower odds of in-hospital mortality among patients ≥70 years old (aOR = 0.82, 95% CI: 0.75–0.89, *p* < 0.001), males (aOR = 0.79, 95% CI: 0.72–0.88, *p* < 0.001), and both obese (aOR = 0.89, 95% CI: 0.79–0.99, *p* = 0.039) and non-obese (aOR = 0.79, 95% CI: 0.71–0.88, *p* < 0.001) patients. In addition, OSA was consistently and significantly associated with increased risk of overall complications among all subgroups (Table 4).

**Table 4:** Stratified analysis on the associations between OSA, in-hospital mortality, prolonged LOS, and complications by age, sex, and obesity status.

Stratum	OSA	In-hospital mortality		Prolonged LOS		Complications, any	
		aOR (95% CI)	*p*-value	aOR (95% CI)	*p-*value	aOR (95% CI)	*p*-value
Age, years							
< 70	Yes vs. No	0.89 (0.76, 1.05)	0.179	1.03 (0.93, 1.13)	0.565	1.30 (1.20, 1.41)	**<0.001**
≥ 70	Yes vs. No	0.82 (0.75, 0.89)	**<0.001**	0.95 (0.88, 1.02)	0.149	1.30 (1.22, 1.40)	**<0.001**
Sex							
Male	Yes vs. No	0.79 (0.72, 0.88)	**<0.001**	1.06 (0.98, 1.14)	0.126	1.27 (1.19, 1.36)	**<0.001**
Female	Yes vs. No	0.91 (0.80, 1.03)	0.142	1.09 (1.00, 1.20)	0.055	1.35 (1.24, 1.47)	**<0.001**
Obesity							
No	Yes vs. No	0.79 (0.71, 0.88)	**<0.001**	1.00 (0.91, 1.09)	0.991	1.31 (1.22, 1.42)	**<0.001**
Yes	Yes vs. No	0.89 (0.79, 0.99)	**0.039**	0.97 (0.89, 1.04)	0.373	1.29 (1.20, 1.39)	**<0.001**

OR, odds ratio; aOR, adjusted odds ratio; CI, confidence interval; LOS, length of stay.

Variables which were significant in univariate regression were adjusted in multivariable models.

*p-values < 0.05 are shown in bold.*

**Table 5:** Stratified analysis on the associations between OSA and specific complications by age, sex, and obesity status.

Subgroup	OSA	Heart failure		CVA		Arrhythmia		Respiratory failure	
		aOR (95% CI)	*p*-value	aOR (95% CI)	*p*-value	aOR (95% CI)	*p*-value	aOR (95% CI)	*p*-value
Age, years									
< 70	Yes vs. No	2.01 (1.76, 2.30)	**<0.001**	0.75 (0.56, 1.00)	0.053	1.13 (0.93, 1.38)	0.209	1.24 (1.15, 1.33)	**<0.001**
≥ 70	Yes vs. No	1.60 (1.48, 1.73)	**<0.001**	0.70 (0.60, 0.81)	**<0.001**	1.19 (1.07, 1.33)	**0.001**	1.17 (1.11, 1.25)	**<0.001**
Sex									
Male	Yes vs. No	1.66 (1.53, 1.81)	**<0.001**	0.69 (0.58, 0.82)	**<0.001**	1.14 (1.01, 1.28)	**0.031**	1.15 (1.09, 1.22)	**<0.001**
Female	Yes vs. No	1.79 (1.60, 1.99)	**<0.001**	0.76 (0.60, 0.96)	**0.019**	1.28 (1.09, 1.51)	**0.003**	1.28 (1.18, 1.38)	**<0.001**
Obesity									
No	Yes vs. No	1.62 (1.48, 1.78)	**<0.001**	0.67 (0.56, 0.80)	**<0.001**	1.27 (1.12, 1.44)	**<0.001**	1.19 (1.11, 1.28)	**<0.001**
Yes	Yes vs. No	1.80 (1.64, 1.98)	**<0.001**	0.78 (0.64, 0.96)	**0.021**	1.09 (0.95, 1.25)	0.236	1.20 (1.12, 1.28)	**<0.001**

OR, odds ratio; aOR, adjusted odds ratio; CI, confidence interval; CVA, cerebrovascular accident.

Variables which were significant in univariate regression were adjusted in multivariable models.

*p-values < 0.05 are shown in bold.*

For specific complications, OSA was significantly associated with heart failure and respiratory failure in all subgroups, and arrhythmia in most subgroups. Further, OSA was significantly associated with lower likelihood of CVA among most subgroups (Table 5).

## 4 Discussion

The study used data from the US NIS database 2020, to investigate the relation between OSA and inpatient outcomes of COVID-19 infection. Overall, OSA was associated with an elevated risk of overall complication rates, irrespective of age, sex, or obesity status. Among specific complications, OSA is associated with increased risk of respiratory failure, heart failure, and arrythmia in most patient subgroups; however, it appears to be associated with decreased likelihood of CVA. Interestingly, OSA seems to be associated with a reduced risk of in-hospital mortality in patients ≥70 years of age, and males but not females. These results indicate that OSA plays a complex yet significant role in influencing the outcomes of COVID-19 patients. This complexity underscores the need for a customized approach in the management and care of these patients, taking into account the unique interplay between OSA and COVID-19. Nevertheless, it should be noted that since the NIS is a hospital database where only hospitalized cases are captured, the study population consisted solely of hospitalized COVID-19 patients, limiting the applicability of the findings to the general population, particularly those with milder cases who did not require hospitalization. That being said, the results can only be generalized to individuals requiring hospitalization within the US healthcare system rather than all individuals infected by COVID-19. Further research using community-based cohorts or electronic health records is still needed to clarify OSA’s impact on mild or asymptomatic COVID-19 cases.

It because obvious during the COVID-19 pandemic that while in most persons the disease was self-limited, an infection with COVID-19 could be life-threatening and that patients with comorbid conditions such as COPD and obesity had markedly worse outcomes. Study has also shown that patients with OSA are at increased risk of a severe course of COVID-19 (12–15). However, a study showed that patients with OSA who were adherent to CPAP therapy were less likely to experience a severer course of COVID-19 (16). Quan et al. (13) also reported that persons with OSA have a greater likelihood of contracting COVID-19 and are more likely to require hospitalization. Similarly, Arish et al. (17) reported that patients with a high OSA risk (determined by Epworth Sleeping Scale and Berlin questionnaire) were more likely to develop severe COVID-19 and require hospitalization.

Our results showed that OSA was associated with increased risk of respiratory failure, heart failure, and arrhythmias in patients with COVID-19 requiring hospitalizations. A systematic review and meta-analysis by Hariyanto et al. (10) that included about 54,000 patients with COVID-19 reported that OSA was associated with significantly poorer overall outcomes, and increased risk of mortality, ICU admission, and the need for mechanical ventilation. On the other hand, Mashaqi et al. (11) performed a cohort study, and multivariable analysis did not show OSA was associated with worse COVID-19 outcomes; however, the study only included about 1,700 patients.

An association between OSA and arrhythmia risk has been little studied, especially in patients with COVID-19. Mouram et al. (18) investigated cardiac arrhythmias in patients with COVID-19 and reported that the need for oxygen therapy and computed tomography (CT) severity score were predictors of arrhythmia occurrence. The study, however, did not examine the effect of OSA.

Unexpectedly, our results showed that OSA was associated with a decreased risk of CVA and mortality in patients >70 years old and men. While this finding may be due to unrecognized confounders, the finding is somewhat consistent with several prior studies that indicated OSA may offer a survival advantage during hospitalization for various conditions. For example, Mohananey et al. (19) showed that patients with OSA and a ST-elevation MI had significantly decreased mortality than patients without OSA. Similarly, Agrawal et al. (20) reported that OSA was associated with lower in-hospital mortality of patients with an AMI, after adjusting for various demographic and co-morbid factors. Another study showed that while patients with OSA hospitalized for non-surgical reasons had higher costs and longer LOS, OSA was associated with decreased mortality (21).

While the aforementioned results may seem counterintuitive, there are reasonable, potential explanations. One possible reason for the observed reduction in CVAs and enhanced survival in OSA patients may be because OSA induces intermittent hypoxia, leading to ischemic preconditioning over time. The repeated episodes ischemia, a characteristic of OSA, may protect against further ischemic damage and infarction as the body becomes “preconditioned” to withstand more severe episodes of ischemia/hypoxemia (22, 23). The benefits of this adaptive response might be accentuated in older patients or males, potentially due to variations in comorbidities or the duration of OSA. Another potential explanation is that OSA can lead to alterations in inflammatory and coagulation pathways, potentially offering some protective effect against the hypercoagulability associated with COVID-19 (24). This complex interplay might be more beneficial in reducing the risk of CVA and mortality in specific patient groups, such as older males. Lastly, the most common therapy for OSA is CPAP during sleep, which significantly improve oxygen saturation during sleep, reduces systemic inflammation, and stabilizes blood pressure (16). These benefits might contribute to lowering the risk of CVAs and mortality, especially if patients are compliant with their treatment. Older males may be more likely to adhere to CPAP therapy, or have been on therapy for longer durations, potentially enhancing these postulated protective effects.

While not examined in this study, it is known that some patients who have had a COVID-19 infection experience long-term sequelae. A recent study showed that patients with OSA were more likely to develop long-term complications of the COVID-19 infection (25). Further studies are needed to address this important issue.

Given these findings, future research should further explore the interplay between OSA, COVID-19, and patient outcomes through detailed study designs and mechanistic investigations. Specifically, studies should incorporate severity scores for both conditions, such as the Apnea-Hypopnea Index (AHI) and COVID-19 severity scales, to refine risk assessment. Evaluating CPAP compliance and its role in mitigating complications is also essential. Additionally, investigating biological mechanisms through biomarker analysis, inflammatory profiling, and genetic studies could clarify how intermittent hypoxia, altered coagulation, or chronic inflammation influence disease progression. The long-term impact of COVID-19 in OSA patients remains uncertain, necessitating research into risks for persistent respiratory dysfunction, cardiovascular events, and neurocognitive impairment. Addressing these gaps will enhance risk stratification, provide mechanistic insights, and guide targeted interventions.

## 5 Strengths and limitations

This study, leveraging data from the 2020 NIS (26), boasts strengths such as its large, nationally representative sample size and the use of PSM to minimize confounding factors and thus enhance the reliability of the results. It offers a comprehensive examination of outcomes, and used stratified analysis to examine relations in different patient subgroups. The exclusion of patients with COPD allowed a focused examination of the impact of OSA on COVID-19 outcomes, considering that COPD is strongly associated with adverse COVID-19 outcomes. However, its retrospective nature introduces potential biases. While CPAP therapy was considered as a covariate, the lack of detailed data on OSA severity, duration of CPAP usage, and patient compliance limits our ability to fully account for the effects of OSA management. Similarly, medications prescribe are not collected by the NIS, thus could not be analyzed. The dependence on ICD codes to identify medical conditions might lead to bias. Despite efforts to control for confounding factors, the potential for residual confounding remains, as not all relevant variables may have been considered. Another significant limitation is the absence of data on the severity of both OSA and COVID-19, which could impact the analytic results. Another key limitation is the inherent selection bias within the study population. Because the dataset is an inpatient dataset, the analysis was limited to hospitalized COVID-19 patients and did not account for individuals with COVID-19 who were not admitted to a hospital, such as those with mild or asymptomatic disease. Presumably, the study might also include a disproportionately higher number of patients with severe or multimorbid OSA, while those with less severe forms of the disease may be underrepresented. Consequently, our findings should be interpreted within the context of hospitalized patients in the US healthcare system and should not be extrapolated to all individuals with OSA and COVID-19. Additionally, the lack of post-discharge follow-up information in the dataset prevents the exploration of long-term morbidity and mortality. Lastly, its findings, based on US data, may not fully translate to non-US healthcare settings, suggesting a need for cautious application of these insights globally.

## 6 Conclusion

In conclusion, this study found that in patients with COVID-19, OSA is associated with an increased risk of several complications, including respiratory failure, heart failure, and arrhythmia, across various patient groups. On the other hand, OSA appears to correlate with a decreased risk of CVAs during COVID-19 hospitalization and reduced in-hospital mortality in males and in patients ≥70 years old. These findings highlight the complexity of the impact of OSA on COVID-19, emphasizing the importance of individualized patient management that considers the intricate interplay between these conditions. As the study focused on hospitalized patients, the findings may not apply to individuals with mild or asymptomatic COVID-19. Future research should aim to include community-based cohorts to assess the broader impact of OSA on COVID-19, including individuals who do not require hospitalization. Prospective studies incorporating OSA severity metrics, CPAP adherence, and long-term outcomes would further refine our understanding of this association.

## Data Availability

The original contributions presented in the study are included in the article/Supplementary material, further inquiries can be directed to the corresponding author.
