# Supplementary material for: Impact of obstructive sleep apnea on inpatient outcomes of COVID-19: a propensity-score matching analysis of the US Nationwide Inpatient Sample 2020
Source: Front Med (Lausanne). 2025 Mar 20;12:1472176. doi: 10.3389/fmed.2025.1472176 (PMC11965585; doi:10.3389/fmed.2025.1472176)
Supplement: Supplementary file 1 [file Table_1.docx]

**Supplementary Table S1. ICD 10 diagnostic codes used in the study**

| Condition | ICD-10-CM |
| --- | --- |
| Acute cholecystitis | K81 |
| Acute pancreatitis | K85 |
| Acquired Immunodeficiency Syndrome | B20.x-B22.x, B24.x |
| Any malignancy | C00 -C26, C30 -C34, C37-C41, C43, C45-C58, C60-C76, C81-C85, C88, C90-C97, C77-C80 |
| Any malignancy, including lymphoma and leukemia, except malignant neoplasm of skin | C00.x-C26.x, C30.x-C34.x, C37.x-C41.x, C43.x, C45.x-C58.x, C60.x-C76.x, C81.x-C85.x, C88.x, C90.x-C97.x |
| Arrhythmia | I49, I47 |
| Atrial fibrillation | I48.2, I48.91 |
| Cerebrovascular disease | G45.x, G46.x, H34.0, I60.x-I69.x |
| Chronic kidney disease | I12.0, I13.1, N03.2-N03.7, N05.2-N05.7, N18, N19, N25.0, Z49.0-Z49.2, Z94.0, Z99.2 |
| Chronic pulmonary disease | J40 -J47, J60-J67, J68.4, J70.1, J70.3 |
| Congestive heart failure | I09.9, I11.0, I13.0, I13.2, I25.5, I42.0, I42.5-I42.9, I43.x, I50.x, P29.0 |
| COVID-19 infection | U07.1, U00, U49, U50, U85, J1282 |
| Cerebrovascular accident | I60, I61, I63, I69, DXCCSR_CIR020>0 |
| Dementia | F00.x-F03.x, F05.1, G30.x, G31.1 |
| Diabetes | E10-E14 |
| Diabetes with chronic complication | E10.2-E10.5, E10.7, E11.2-E11.5, E11.7, E12.2-E12.5, E12.7, E13.2-E13.5, E13.7, E14.2-E14.5, E14.7 |
| Diabetes without chronic complication | E10.0, E10.l, E10.6, E10.8, E10.9, E11.0, E11.1, E11.6, E11.8, E11.9,E12.0, E12.1, E12.6, E12.8, E12.9, E13.0, E13.1, E13.6, E13.8, E13.9, E14.0, E14.1, E14.6, E14.8, E14.9 |
| Disseminated intravascular coagulation | D65, O46.02, O46.029, O45.029, O45.02 |
| Encephalitis/meningitis | G00-G09 |
| Gastrointestinal hemorrhage | K92.2, K62.5, K28.2-9, K91.62 |
| Heart failure | I50, I09.81, I97.13, I11.0 |
| Hemiplegia or paraplegia | G04.1, G11.4, G80.1, G80.2, G81.x, G82.x, G83.0-G83.4, G83.9 |
| Hemophagocytic lymph histiocytosis | D76.1, D76.2 |
| Hypertension | I10-I16, I1A |
| Influenza A and B | J10.XX, J11.XX, J09.XX |
| Ischemic heart disease | I20-I25 |
| Mechanical ventilation | Z99.12 PCS: 5A1935Z, 5A1945Z, 5A1955Z |
| Metastatic solid tumor | C77.x-C80.x |
| Mild liver disease (without portal hypertension, includes chronic hepatitis) | B18.x, K70.0-K70.3, K70.9, K71.3-K71.5, K71.7, K73.x, K74.x, K76.0, K76.2-K76.4, K76.8, K76.9, Z94.4 |
| Moderate or severe liver disease | I85.0, I86.4, K70.4, K71.1, K72.1, K72.9, K76.5, K76.6, K76.7 |
| Moderate or severe renal disease | I12.0, I13.1, N03.2-N03.7, N05.2-N05.7, N18.x, N19.x, N25.0, Z49.0-Z49.2, Z94.0, Z99.2 |
| Myocardial infarction | I21.x, I22.x, I25.2 |
| Obesity | E66.0, E66.1, E66.2, E66.8, E66.9  CM_OBESE=1 |
| Obstructive sleep apnea | G47.33 |
| Peptic ulcer disease | K25.x-K28.x |
| Peripheral vascular disease | I70.x, I71.x, I73.1, I73.8, I73.9, I77.1, I79.0, I79.2, K55.1, K55.8, K55.9, Z95.8, Z95.9 |
| Respiratory failure | J95.1-J95.8, J96 |
| Rheumatic disease | M05.x, M06.x, M31.5, M32.x-M34.x, M35.1, M35.3, M36.0, |
| Rheumatic disease | M05.x, M06.x, M31.5, M32.x-M34.x, M35.1, M35.3, M36.0 |
| Severe liver disease | I85.0, I86.4, K70.4, K71.1, K72.1, K72.9, K76.5, K76.6, K76.7 |
| Smoking | Z71.6 Z72.0, Z86.43, Z87.891, F17, O99.33, T65.2 |
| Venous thromboembolism | I26.0, I26.9, I80.0 -I80.3, I80.8, I80.9, I81, I82, O08.2, O22.3, O87.1, O88.2 |

CM, clinical modification; PCS, procedure codes.
